# Is Non-Operative Management With Closed Reduction and Casting a Safe Option for Gartland Type II Supracondylar Fractures? A Review of the Literature

**DOI:** 10.7759/cureus.95778

**Published:** 2025-10-30

**Authors:** Sofia Bitsios, Ramy Elemam, Dominique Dennis

**Affiliations:** 1 Orthopaedics, Norfolk and Norwich University Hospital, Norwich, GBR; 2 Orthopaedics, Broomfield Hospital, Chelmsford, GBR

**Keywords:** gartland type ii, non-operative management, paediatric supracondylar fracture, supracondylar humeral fracture, type 2 gartland

## Abstract

Gartland type II supracondylar humerus fractures represent a spectrum of injuries in children where treatment remains controversial. Although current guidelines favour operative fixation, emerging evidence suggests that non-operative treatment may be appropriate for selected cases.

This review summarises current literature on the safety and efficacy of non-operative management for Gartland type II fractures and identifies radiographic and clinical predictors of treatment success or failure. Retrospective and prospective studies comparing operative and non-operative approaches were analysed, with particular attention to predictors of reduction loss and the influence of the IIA/IIB Wilkins-modified subclassification.

Success rates for non-operative treatment ranged from 70% to 90% in carefully selected cases, with loss of reduction occurring in 10%-25%, especially in fractures with extension deformity, sagittal obliquity or metaphyseal fragmentation. Subclassification improved prognostic accuracy: type IIA fractures were generally stable, while type IIB fractures had higher failure rates. However, these appeared best combined with other valuable radiographic predictors of stability, including the hourglass angle, humerocapitellar angle and anterior humeral line index. When alignment was maintained, functional outcomes were comparable between operative and non-operative groups.

In conclusion, non-operative management with closed reduction and casting is safe for select Gartland type II fractures with favourable radiographic features. However, close follow-up with early repeat imaging is essential, and standardised selection criteria remain lacking. Future multicentre prospective studies are needed to validate radiographic thresholds and refine treatment guidelines.

## Introduction and background

Supracondylar humerus fractures are among the most common paediatric elbow injuries [1]. They are typically classified using the Gartland system. Gartland type II fractures are defined by displacement with an intact posterior cortex, representing an intermediate category between non-displaced (type I) and completely displaced (type III) injuries [2]. However, this intermediate category encompasses a wide spectrum of fractures. Therefore, the Wilkins-modified Gartland classification was devised to further sub-classify type II fractures into type IIA (those without rotational or translational deformity) and type IIB (those with rotational and/or translational deformity) [1]. This distinction was intended to aid clinicians in determining appropriate management, as it has been proposed that type IIA fractures may represent a more stable subset of type II supracondylar fractures and could be managed non-operatively. Conversely, type IIB fractures may represent an inherently unstable subset of fractures and may therefore be better managed operatively, usually with closed reduction and percutaneous pinning (CRPP) [1]. The implications of this subclassification potentially include reduced healthcare costs, reduced operative burden and fewer operative complications. It could also reduce patient and family distress and parental time off work if a larger proportion of fractures were determined safe for non-operative management. However, the Wilkins-modified Gartland classification shows inter-surgeon variability in determining fracture subtype, and despite its existence, the management of type II supracondylar fractures remains widely debated.

Current UK guidance, the British Orthopaedic Association Standards for Trauma (BOAST) guidelines, frame displaced supracondylar fractures as injuries requiring urgent orthopaedic evaluation and often surgical stabilisation, emphasising prompt neurovascular assessment and pin fixation where indicated, although they do not specify management by Gartland subtype [3]. The American Academy of Orthopaedic Surgeons (AAOS) guidelines recommend CRPP for all displaced type II and III fractures [4]. These positions reflect concern for stability and the predictable success of fixation.

However, operative management involves anaesthesia, theatre time, and potential complications, including pin site infections and iatrogenic nerve injuries [2]. It also usually requires an inpatient stay and a surgeon who is trained in performing the procedure. In contrast, successful non-operative management could reduce operative burden, reduce costs and may reduce distress for children and families. This review evaluates whether non-operative management is safe for Gartland type II fractures, which subgroups, if any, can be managed conservatively and which radiographic parameters could help to guide decision-making.

## Review

Methods

Search Strategy

A systematic search of PubMed, Embase and the Cochrane Library was undertaken for studies published between January 2000 and September 2025. Search terms included ‘Gartland type 2’, ‘Gartland type II’, ‘non-operative management’, ‘conservative management’, and ‘supracondylar fracture’, combined using Boolean operators (AND/OR) where appropriate. Only studies written in, or translated into, English were included. Reference lists of all eligible studies were also manually screened to identify additional relevant publications.

Eligibility Criteria

Studies were eligible if they involved paediatric patients with Gartland type II supracondylar humeral fractures and assessed outcomes of non-operative management, either alone or compared with surgical fixation. Studies examining predictors of reduction loss or subclassifications (IIA/IIB) were also included. Eligible designs comprised prospective or retrospective cohort studies, case-control studies, clinical trials and systematic reviews. Case reports, conference abstracts, non-peer-reviewed papers and studies unrelated to type II fractures were excluded.

Study Selection

**Figure 1:**
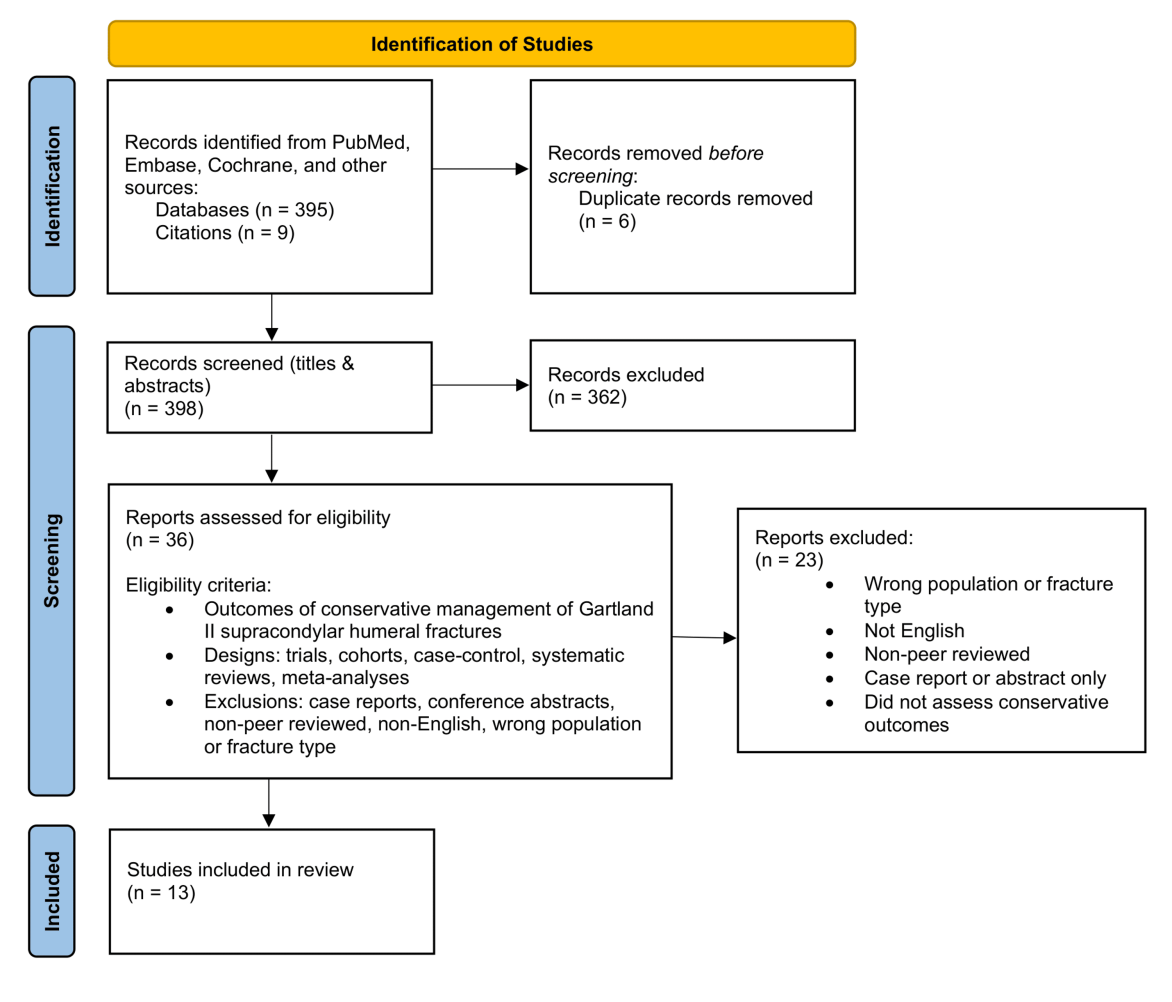
PRISMA diagram outlining identification of studies for inclusion in the literature review

The database search yielded 398 papers. Following title and abstract screening, 36 studies were selected for full-text review. Three independent reviewers (S.B., D.D., and R.E.) screened all studies at each stage, and any disagreements were resolved through discussion until consensus was reached. Thirteen studies met all inclusion criteria and were included in the final analysis. The study selection process is summarised in the PRISMA diagram (Figure 1).

Quality Appraisal

Methodological quality was assessed using the Risk of Bias in Non-randomised Studies of Interventions (ROBINS-I) tool [5], applied independently by two reviewers. Discrepancies were resolved by consensus. Most studies demonstrated a moderate overall risk of bias, reflecting their retrospective design and potential confounding. Two comparative studies were rated as having a serious risk of bias due to selection and confounding factors. The results of the quality assessment are presented in Table 1.

**Table 1:** Risk of bias assessment using ROBINS-I ROBINS-I: Risk of Bias in Non-randomised Studies of Interventions.

Study (Author, Year)	Study Design	Risk of Bias (ROBINS-I) [5]
Fitzgibbons et al., 2011 [6]	Retrospective case-control	Moderate
Yildirim et al., 2022 [7]	Retrospective cohort	Moderate
Zhu et al., 2024 [8]	Retrospective comparative (non-operative vs operative)	Serious
Ojeaga et al., 2020 [9]	Retrospective cohort	Moderate
McCartney et al., 2022 [10]	Retrospective cohort	Moderate
Sanders et al., 2023 [11]	Prospective cohort (non-operative vs operative)	Moderate
Camus et al., 2011 [12]	Retrospective case series	Moderate
Güneş et al., 2024 [13]	Retrospective comparative (non-operative vs operative)	Serious
Pierantoni et al., 2020 [14]	Retrospective case series	Moderate
Şişman et al., 2022 [15]	Retrospective case-control	Moderate
Amaral et al., 2024 [16]	Retrospective case series	Moderate
Teo et al., 2020 [17]	Retrospective cohort	Moderate
Leung et al., 2018 [18]	Retrospective cohort	Moderate

Results

Success Rates of Non-operative Care

Across multiple cohorts, approximately 70%-80% of carefully selected Gartland type II supracondylar fractures treated with closed reduction and casting maintained acceptable alignment without requiring conversion to surgery [6-10]. Reported conversion rates to percutaneous pinning ranged widely, from as low as 4%-9% in series focusing on type IIA fractures, up to around 20%-25% in mixed cohorts that also included type IIB injuries [6,8-11]. A detailed breakdown of success rates and functional outcomes for each included study is presented in Table 2.

**Table 2:** The success rate of conservative management and functional outcomes for all studies evaluated in this review CRPP: Closed reduction and percutaneous pinning; AHL: Anterior humeral line.

Study (Author, Year)	Study Type	Sample Size	Fracture Subtype	Success Rate (Conservative)	Conversion to CRPP	Functional Outcomes
Fitzgibbons et al., 2011 [6]	Retrospective case-control	61 (Type II)	Mixed (all type II)	80% (49/61 maintained reduction)	11% (7/61)	Not reported (focus on radiographic stability)
Yildirim et al., 2022 [7]	Retrospective	103 (Type II)	Mixed (IIA and IIB)	79% (81/103) maintained reduction	21% (22/103)	Not reported (studied radiographic risk factors)
Zhu et al., 2024 [8]	Retrospective comparative	142 (Type II)	Mixed (IIA and IIB)	~80% (19.8% loss of reduction)	19.80%	Comparable to surgical group; faster ROM recovery in conservative group
Ojeaga et al., 2020 [9]	Retrospective	77 (Type IIA)	IIA only	76.6% (59/77)	23.4% (18/77)	Not reported (identified radiographic predictors)
McCartney et al., 2022 [10]	Retrospective	54 (Type IIA)	IIA only	70% (38/54)	30% (16/54)	Not reported (focused on radiographic differences)
Sanders et al., 2023 [11]	Prospective cohort	99 (Type IIA)	IIA only	90% (41/45)	9% (4/45)	No difference versus CRPP group (Flynn criteria good-excellent; similar QuickDASH/Mayo scores at 6–24 months)
Camus et al., 2011 [12]	Retrospective	155 (Type II)	Mixed (all type II)	100% (no cases required surgery)	0%	No functional data – 80% had residual extension deformity on X-ray (AHL not through middle capitellum) (clinical significance unclear)
Güneş et al., 2025 [13]	Retrospective comparative	55 (23 non-op vs 32 op)	Mixed (IIA and IIB)	95–96% (22/23 in conservative group)	~4% (1/23)	Equivalent mid-term functional scores (Mayo Elbow, QuickDASH) between conservative and surgical groups
Pierantoni et al., 2020 [14]	Retrospective	31 (Type II)	Mixed (IIA and IIB)	84% (26/31)	16% (5/31)	Flynn criteria: 100% good/excellent; QuickDASH ~0 at final follow-up (no functional impairment despite mild residual varus)
Şişman et al., 2022 [15]	Retrospective case-control	87 (52 IIA, 35 IIB)	IIA vs IIB analysed	IIA: ~94% (49/52); IIB: ~74% (26/35)	IIA: ~6%; IIB: ~26% (loss of reduction rates)	No difference in final clinical or radiographic outcomes between IIA and IIB if reduction maintained; outcomes of delayed ('rescue') pinning were equivalent to immediate pinning
Amaral et al., 2024 [16]	Retrospective	~21 (Type IIA)	IIA only	~95% (vast majority maintained alignment)	4.70%	Not reported (focused on 1-week alignment check and early intervention rates)

Fitzgibbons et al. (2011) reported maintenance of alignment in 49 out of 61 cases (80%) of Gartland type II supracondylar fractures treated with closed reduction and casting in the emergency department. Twelve patients showed loss of reduction on follow-up radiographs, with a total of seven ultimately undergoing delayed CRPP. The remaining five were treated non-operatively despite some loss of alignment [6]. Yildirim et al. (2022) also examined closed reduction followed by casting or splinting of type II fractures in the emergency department. They defined loss of reduction as >12° change in Baumann’s angle or >10° change in the shaft-condylar angle. Out of 103 patients, 81 (79%) maintained adequate alignment without surgery [7]. Zhu et al. (2024) directly compared closed reduction and casting versus primary CRPP for Gartland type II fractures in 142 children. Loss of reduction occurred in 19.8% of conservatively managed cases. Notably, when broken down by subtype, type IIB fractures had a 41.3% risk of losing reduction compared to only 5.3% for type IIA fractures [8].

**Figure 2:**
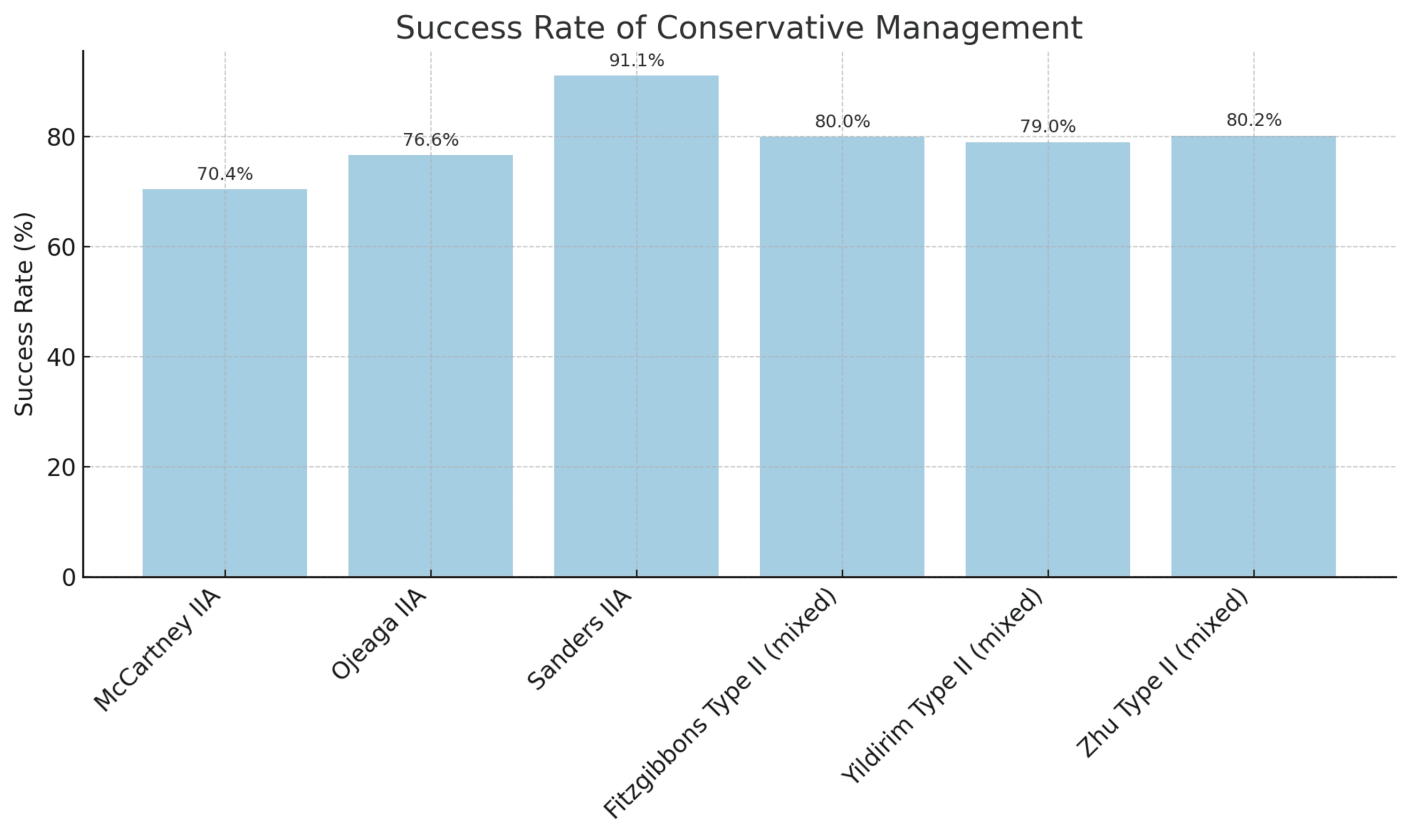
Success rate of conservative management of Gartland II supracondylar humeral fractures, broken down by study and fracture subtype (type IIA only or mixed groups containing both type IIA and IIB fractures)

Ojeaga et al. (2020) treated 77 children with type IIA fractures using closed reduction and casting. Fifty-nine of these (76.6%) maintained alignment, while the other 18 patients required delayed CRPP. They found that performing the reduction under procedural sedation in the emergency department was strongly associated with success (83% success with sedation versus 56% without) [9]. McCartney et al. (2022) likewise used conscious sedation for closed reduction of type IIA fractures in the emergency department. Non-operative management was successful in 38 of 54 patients (70%), with 16 patients (30%) requiring conversion to pinning [10]. Sanders et al. (2023) reported on a prospective cohort of type IIA fractures, showing successful conservative management in 41 out of 45 patients (91%). Four patients (9%) required conversion to CRPP by the first follow-up visit [11]. Figure 2 illustrates the range of success rates of conservative management reported by each study, stratified by whether the cohort included only type IIA fractures or a mix of IIA and IIB injuries.

Contrary to the above studies, Camus et al. (2011) reviewed 155 children with Gartland type II fractures initially managed with closed reduction and casting and found that none of these patients required conversion to surgical treatment. However, at final follow-up (average 5.3 months), 80% of the patients’ radiographs showed that the anterior humeral line did not bisect the middle third of the capitellum, and the mean humerocapitellar angle (HCA) was 23.8° - findings indicative of a persistent extension deformity. Furthermore, 47% of patients had a Baumann’s angle outside the normal range, and 44% showed a Griffet index < 3 (suggesting poor rotational alignment). In summary, the majority of Gartland II fractures in this series demonstrated some degree of residual deformity on radiographs when treated non-operatively. The clinical significance of these radiographic abnormalities remains unclear, as Camus et al. did not assess functional outcomes in this cohort [12].

Functional Outcomes

Several studies evaluated functional outcomes following non-operative management with closed reduction and casting. They showed that when alignment was maintained, medium-term functional results (as assessed by Flynn’s criteria, QuickDASH scores, Mayo Elbow scores, and range of motion) were generally comparable to those achieved with primary surgical fixation [8,11,13-14]. For example, Sanders et al. (2023) found that the conservatively managed group had slightly more residual extension visible on six-month radiographs compared to the operatively managed group. However, this subtle difference did not translate into any significant difference in clinical function or patient-reported outcome measures at six-month and 24-month follow-ups [11]. Similarly, Pierantoni et al. (2020) noted a mild residual cubitus varus deformity on radiographs in the non-operative group at final follow-up, yet this did not cause functional impairment: All patients had good or excellent results by Flynn’s criteria, and the average QuickDASH score was essentially zero, indicating no disability [14]. Interestingly, Zhu et al. (2024) reported that patients managed conservatively regained elbow range of motion faster than those who underwent pinning, with no difference in overall fracture-healing time between the groups [8]. Across all studies, the rates of major complications (such as vascular compromise, permanent nerve palsy, or clinically significant malunion deformities) were very low, regardless of whether the fracture was managed with casting or surgery [8,11,13-14].

Radiographic Predictors of Failure

Multiple radiographic factors were proposed as predictors of which fractures might lose alignment after closed reduction and casting. Both Fitzgibbons et al. (2011) and McCartney et al. (2022) found that a pronounced extension deformity on the lateral injury film was strongly associated with failure of closed reduction. In practical terms, if the capitellum lay entirely anterior to the anterior humeral line (AHL) - indicating a disrupted anterior cortical alignment - the risk of losing reduction was significantly higher (p < 0.01; Fitzgibbons et al.) [6]. McCartney et al. noted that this held true on both the initial injury radiograph and the post-reduction film. In their series, fractures that ultimately remained stable in a cast had a mean extension angle of 13.2° on the injury film and 3.0° after reduction, whereas those that went on to fail casting had a mean extension of 19.8° initially and 10° after reduction (a significant difference, p = 0.008) [10]. They also quantified the anterior humeral line’s position (the ‘AHL index’) and found that a lower AHL index - meaning the capitellum was positioned far anterior relative to the humeral shaft - correlated with a higher likelihood of loss of reduction, particularly on the post-reduction radiograph [10]. In addition, Fitzgibbons et al. observed that a wide upper arm soft-tissue shadow on the post-reduction X-ray (suggesting substantial swelling or a loosely fitting cast) was a significant risk factor for loss of reduction (p = 0.016) [6]. Based on these findings, the authors of those studies recommended considering primary pinning in cases with a large extension gap or significant swelling, even if a closed reduction is initially achieved.

Yildirim et al. (2022) identified two other radiographic risk factors for casting failure. High sagittal-plane obliquity of the fracture (i.e., a steep fracture line on the lateral view) was associated with approximately 3.2-fold higher odds of loss of reduction (p < 0.05), and the presence of metaphyseal fragmentation at the fracture site was associated with about a 6.5-fold increase in odds of failure (p < 0.01) [7]. In their analysis, the Gartland subclassification (type IIA versus IIB) was not itself a significant independent predictor of failure, suggesting that fracture morphology features may be more important than the subtype label in predicting stability [7].

Some studies specifically compared outcomes of type IIA versus type IIB fractures managed non-operatively. Overall, type IIB fractures (those with some translational or rotational deformity) showed substantially higher secondary displacement rates than type IIA fractures. For example, Zhu et al. (2024) observed a loss of reduction in 41.3% of conservatively treated type IIB fractures, versus only 5.3% of type IIA fractures (approximately an eight-fold difference) [8]. Şişman et al. (2022) similarly reported that loss of reduction was roughly five times more likely in type IIB than in type IIA injuries (nine cases versus three cases in their series, p ≈ 0.01) [15]. Notably, Şişman et al. also found that if a type IIB fracture was initially reduced and remained stable through the first week of casting, the longer-term outcomes of non-operative management were equivalent to those of type IIA fractures. They additionally showed that patients who required delayed pinning after an initial attempt at casting ultimately had final outcomes comparable to those who had been pinned immediately, without an increase in complications or deformity due to the brief delay in surgery [15]. In contrast to the above, Yildirim et al. (2022) did not find the Gartland subtype to be a significant factor for predicting loss of reduction in their cohort, as noted earlier [7].

Several novel measurements on post-reduction radiographs were also proposed as tools to gauge stability. Ojeaga et al. (2020) introduced the concept of the hourglass angle (HGA), defined as the angle of the ‘waist’ of the distal humerus on a lateral view (formed by the intersection of lines along the anterior cortices of the distal humeral columns). They found that cases with a larger post-reduction HGA were more likely to maintain alignment in a cast. In their data, the mean HGA after reduction was 169.4° in successful cases versus 163.2° in failures (with an average ‘normal’ HGA in uninjured arms around 177.8°) - a statistically significant difference [9]. Ojeaga et al. reported excellent inter-observer reliability for measuring the HGA. By contrast, the HCA - essentially the initial extension angle of the fracture - had poor inter-observer reliability in their study, and its predictive value was less clear. While the fractures that did well non-operatively tended to have a higher initial HCA (indicating a less extended fracture position), this parameter alone was inconsistent and not as reliable for decision-making [9]. Notably, Ojeaga et al. also observed that if the extension deformity was not adequately corrected by the reduction (as measured by a persistent gap between the AHL and the capitellum), the fracture was more likely to displace later (i.e., maintaining a significant anterior gap after reduction was a warning sign for failure) [9].

Sanders et al. (2023) measured radiographic alignment at follow-up and reported an average hourglass angle of 176.9° at six months post-injury in cases that had been successfully treated non-operatively. However, their study did not formally analyse specific post-reduction radiographic parameters as predictors of success, so the utility of HGA in their cohort was not directly assessed [11]. On the other hand, Güneş et al. (2025) provided a contrasting perspective on the HCA: They reported that a higher HCA (which corresponds to a more extended initial fracture position) was associated with a more unstable injury that eventually required surgical fixation in their series. This finding contrasts with Ojeaga’s observations and suggests that different patient populations or measurement techniques might yield conflicting results regarding which radiographic thresholds indicate instability [13]. Table 3 summarises the various radiographic parameters examined in each study, along with the authors’ conclusions about their predictive value.

**Table 3:** Radiographic parameters assessed per study and author recommendations

Study (Author, Year)	Study Type	Sample Size	Fracture Subtype	Radiographic Predictor(s) Assessed	Predictive Value/Risk Estimate	Conclusion on Predictor’s Utility
Fitzgibbons et al., 2011 [6]	Retrospective case-control	61 (type II)	Mixed (all type II)	– Extension deformity (capitellum anterior to AHL on lateral)	– Marked extension (AHL not intersecting capitellum) strongly predicted failure (p < 0.01).	Fractures with a large extension gap (disrupted anterior alignment) or notable swelling are at high risk of losing reduction. The authors recommend considering primary pinning in such cases.
				– Upper arm soft-tissue shadow (swelling/cast fit)	– Wide upper arm soft-tissue shadow on post-reduction film was a significant risk factor (p = 0.016).	
Yildirim et al., 2022 [7]	Retrospective	103 (type II)	Mixed (IIA and IIB)	– Sagittal-plane obliquity (fracture angle in lateral plane)	– High sagittal obliquity: ~3.2× higher odds of loss of reduction (p < 0.05).	Fracture morphology was strongly predictive: high extension (obliquity) and metaphyseal fragment gaps independently increased failure risk. These features may be more useful than the Gartland subclass alone for guiding treatment.
McCartney et al., 2022 [10]	Retrospective	54 (type IIA)	IIA only	– Fracture extension angle (degree of extension on lateral X-ray)	– Failed cases had significantly greater extension deformity: e.g. non-op success group vs failure group mean extension 3° vs 10° after reduction (p = 0.008 for difference).	A large residual extension angle (capitellum positioned anteriorly) on injury or post-reduction films strongly predicts casting failure. A markedly low AHL index after reduction indicates instability, warranting early pinning consideration.
				– Anterior humeral line (AHL) index	– Lower AHL index (capitellum far anterior) on post-reduction films correlated with loss of reduction.	
Ojeaga et al., 2020 [9]	Retrospective	77 (type IIA)	IIA only	– Humerocapitellar angle (HCA) (initial extension angle)	– Successful cases had higher initial HCA and larger post-reduction HGA on average. Normal HGA ~177.8; cases maintaining reduction had post-reduction HGA ~169.4 vs 163.2 in failures (significant difference).	A higher post-reduction HGA was a reliable indicator of a stable reduction. Children who eventually needed surgery showed a failure to restore adequate HGA (and often had lower initial HCA) after reduction. HGA is a useful measure for monitoring, whereas HCA proved less reliable.
				– ‘Hourglass’ angle (HGA) (angle of distal humerus waist on lateral)	– Inter-observer reliability: excellent for HGA and poor for HCA.	
Zhu et al., 2024 [8]	Retrospective comparative	142 (type II)	Mixed (IIA and IIB)	– Gartland subtype (IIA vs IIB)	– Type IIB fractures had a 41.3% loss-of-reduction rate vs only 5.3% for type IIA fractures (≈8× higher, p < 0.001).	Wilkins’ subclassification was strongly prognostic in this cohort: Type IIB injuries were far more prone to secondary displacement than type IIA. Fractures without rotational/translational deformity (IIA) had a high likelihood of successful casting.
Şişman et al., 2022 [15]	Retrospective case-control	87 (52 IIA, 35 IIB)	IIA vs IIB groups	– Gartland subtype (IIA vs IIB)	– Loss of reduction was ~5× more likely in type IIB vs type IIA (9 vs 3 cases; p≈0.01). All losses occurred by the one-week follow-up (none thereafter).	Gartland IIB fractures are inherently less stable than IIA, though if a closed reduction is maintained through the first week, longer-term outcomes match those of IIA. Early re-check is critical; notably, patients needing delayed pinning (after initial casting) had final outcomes equivalent to those pinned immediately.
Güneş et al., 2025 [13]	Retrospective comparative	55 (type II)	Mixed (IIA and IIB)	– Humerocondylar angle (HCA) (lateral extension angle)	– HCA was significantly higher in fractures that required surgical fixation compared to those successfully treated conservatively (difference driven largely by type IIB cases). (i.e. operative group had a larger lateral angle than non-op group.)	A surprisingly higher HCA (less flexed position) was associated with an unstable fracture in this series. This finding contrasts with some prior studies and suggests that radiographic parameters should be interpreted alongside clinical judgement when selecting type II cases for surgery.

Timing of Failure and Follow-Up Needs

The first week after reduction was consistently identified as the highest-risk period for loss of alignment across the included studies [9,15-16]. These studies all support early follow-up with repeat radiographs within about seven days of post-reduction to detect any fracture displacement and allow prompt conversion to operative management if needed. Importantly, prompt conversion to CRPP upon early loss of reduction has been shown to lead to equivalent outcomes compared to immediate pinning at presentation, with no long-term penalty for the brief delay in surgery [15].

Discussion

Success of Non-operative Management

The evidence assembled in this review suggests that non-operative management can be a safe and effective option for a carefully selected subset of Gartland type II supracondylar fractures. In particular, fractures without significant displacement or rotation (especially the so-called type IIA injuries) or those meeting certain favourable radiographic criteria may be suitable for closed reduction and casting. Clinically, expanding the use of non-operative treatment in appropriate cases could reduce the number of surgeries required for this common paediatric injury. Potential advantages of successful non-operative management include lower healthcare costs, avoidance of general anaesthesia and surgical risks, and possibly a quicker recovery of elbow motion for the child [2,8]. However, to safely implement this approach, clear and standardised selection criteria are needed to identify which cases can be managed without pins. At present, while the Gartland IIA versus IIB subclassification provides a starting point for stratifying fracture stability, the evidence indicates that this classification alone is not sufficient to guide treatment decisions in every case. This is partly because the subclassification can be applied inconsistently: There is known low inter-observer reliability in distinguishing between type IIA and IIB fractures on radiographs [17,18]. It is also because some fracture characteristics beyond the Gartland definition (such as the degree of obliquity or comminution) play an important role in stability, as highlighted by Yildirim et al. [7].

Many of the studies in this review demonstrated high success rates for conservative management even in cohorts that included some type IIB fractures, indicating that strict classification by itself may not capture all fractures amenable to casting. That said, it is clear that fractures with any rotational or translational malalignment (Gartland type IIB) are, as a group, more prone to secondary displacement than those with purely extension deformity (IIA). This aligns with the conventional understanding that the added instability from rotational components makes reductions harder to hold. For example, both Zhu et al. and Şişman et al. documented significantly higher failure rates in type IIB injuries, reinforcing the idea that the presence of a rotational deformity is a strong risk factor for casting failure [8,15]. On the other hand, one study (Yildirim et al.) did not find the Gartland subtype to be predictive on its own once other factors were accounted for, underscoring that we should not over-rely on the subclassification without considering fracture morphology in detail [7]. Taken together, the modified Gartland classification can serve as an initial guide: A type IIB designation should raise concern and likely lower the threshold for recommending pin fixation. But beyond this, incorporating additional radiographic criteria could improve our ability to select cases for non-operative treatment.

Radiographic Parameters

Several specific radiographic parameters show promise in refining the selection of stable versus unstable fractures. Among these, the hourglass angle (HGA) - a novel measure introduced by Ojeaga et al. - appears to be a useful indicator of reduction quality and fracture stability, with the advantage of excellent inter-rater reliability in their assessment [9]. A sufficiently large HGA after reduction essentially reflects that the distal humeral metaphysis has been brought into an adequately extended (or neutral) alignment relative to the shaft. In Ojeaga’s study, a lower post-reduction HGA (indicating that the ‘waist’ of the distal humerus remained narrow, and the fracture was still in relative extension) was associated with subsequent loss of position, whereas higher HGA values were associated with successful casting outcomes. This parameter, however, has so far been evaluated in only a single retrospective study of 77 patients [9]. Its true predictive value needs confirmation in larger cohorts and ideally in prospective settings before it could be adopted in guidelines. The HCA, another intuitive measure of fracture extension, yielded conflicting results in different studies and also suffered from measurement variability. Ojeaga et al. suggested that a higher initial HCA (a less extended fracture) was associated with successful non-operative treatment, but Güneş et al. conversely reported that higher HCA measurements were seen in fractures that ended up requiring surgery [9,13]. Moreover, Ojeaga’s team found poor inter-observer agreement for HCA measurements, making it a less reliable metric in practice. A high degree of extension deformity in general, however, emerged as an important red flag for instability throughout the literature, whether quantified by the AHL not intersecting the capitellum, by a large anterior gap between the capitellum and humeral shaft on lateral X-ray, or by a steep fracture angle. Multiple authors observed that fractures with a pronounced extension alignment (after reduction) were much more likely to fail casting and ultimately need pins [6,9,10]. This consistent finding suggests that developing a standard way to assess the residual extension (e.g. using the AHL index or the perpendicular distance from the AHL to the capitellum) could be very useful. Any such measurement tool would need to be validated for accuracy and inter-observer consistency, as none of the current studies evaluated how reproducibly different clinicians could measure these angles or distances.

In light of the evidence, certain radiographic and clinical features can be highlighted as clear warnings that a fracture is likely too unstable for casting alone. These higher-risk features, which should prompt strong consideration of immediate surgical fixation, include the presence of rotation or translation (Gartland type IIB status), a markedly large extension angle on initial films, a very oblique fracture line in the sagittal plane, and the presence of metaphyseal comminution or fragmentation at the fracture site [6,7]. Of course, neurovascular status remains paramount in the acute setting for any supracondylar fracture. Any signs of compromised circulation or nerve function should prompt urgent fracture reduction and likely early surgical intervention regardless of radiographic classification [3].

Practical Considerations

If a decision is made to manage a type II supracondylar fracture non-operatively, several practical considerations emerge from this body of research. Rigorous follow-up is essential. The first post-reduction week is the critical window during which secondary displacement is most likely to occur, so an early clinical review with repeat X-rays (often around five to seven days after reduction) is widely recommended [6,8,9,15]. Early identification of a loss of alignment allows for timely ‘rescue’ pinning, and evidence indicates that when such conversion is done promptly, the clinical outcomes are just as good as if the patient had been pinned on day one [15]. Families should be clearly counselled at the outset that there is a significant chance (reported in some series up to roughly one in five) that surgery may ultimately be needed even after an initial attempt at casting. This underscores the importance of attending all follow-up appointments and monitoring the child closely at home for any signs of the cast becoming loose or the fracture shifting.

The likelihood of needing a later operation varies with how strictly patients are selected for casting. In the studies reviewed, the overall rates of loss of reduction (and thus conversion to surgery) ranged roughly from 5% to 25%. The lowest failure rates (on the order of only 5%-10%) were reported in the most favourably selected cases - for instance, purely type IIA fractures reduced under ideal conditions. By contrast, cohorts that included more borderline cases or type IIB injuries saw higher failure rates, approaching 20%-25% in some reports [6,8-11]. Because most of these studies were small and retrospective, these percentages should be interpreted with caution. Larger prospective trials would be needed to get a more precise estimate of the true failure risk when specific selection criteria are applied. Nevertheless, this information can help clinicians and families weigh the risks and benefits of a casting trial.

Several authors also emphasised the role of adequate sedation or anaesthesia during the initial reduction, as it may improve the quality of reduction and thus the chances of success. For example, Ojeaga et al. documented significantly better outcomes when reductions were performed under proper sedation in the emergency department setting [9]. Ensuring the child is comfortable and the muscles are relaxed likely facilitates a more anatomic reduction and allows for a well-moulded cast. However, the availability of paediatric sedation or anaesthesia in the emergency context can vary between institutions, and in some settings, every displaced fracture is reduced in the operating theatre by default. Centres that wish to adopt non-operative management protocols will need to have both the staffing and the follow-up infrastructure to support this approach safely.

Limitations of Included Studies

The majority of the studies reviewed were retrospective, introducing potential for selection bias, unmeasured confounders and inconsistent data collection. Sample sizes were frequently small and varied in fracture subtype composition, with some studies failing to distinguish outcomes between type IIA and IIB injuries. Radiographic criteria such as the hourglass angle, HCA, and AHL index were inconsistently applied, and standardised thresholds for instability have not been established. Inter-observer reliability was rarely assessed, and definitions of ‘loss of reduction’ and treatment success differed between studies. Additionally, while some studies used validated functional outcome measures, others relied solely on radiographic alignment, limiting comprehensive assessment of clinical impact.

Limitations of This Review

This literature review may be limited by the inherent variability of the included studies and the reliance on predominantly retrospective data. Although efforts were made to include both prospective and comparative studies, heterogeneity in study designs, outcome measures and fracture classification may affect the synthesis of findings. Formal meta-analysis was not feasible, and the risk of bias was qualitatively assessed. Furthermore, relevant unpublished data or studies not indexed in the selected databases may have been missed. These limitations restrict the generalisability of the conclusions and highlight the need for future high-quality, prospective research using standardised radiographic and clinical criteria.

Future Considerations

To move forward, high-quality prospective research is needed. In particular, a prospective or randomised controlled trial comparing non-operative versus operative management in Gartland II fractures - using clearly defined radiographic selection criteria - would provide stronger evidence on outcomes. Such studies should ideally be multicentre to increase generalisability and should track not only union and complication rates but also cost-effectiveness and patient-reported outcomes. Given the potential system-level savings of avoiding unnecessary surgery, it would be valuable to know whether a casting approach (with appropriate safeguards) delivers equal long-term results from the patient and family perspective.

Summary

Overall, the findings of this review indicate that there is credible promise for the safe use of non-operative management in selected Gartland type II supracondylar humeral fractures. This evidence stands in some contrast to the current prevailing guidelines. The BOAST guidelines, for example, do not outright forbid non-operative treatment for appropriate cases, but it emphasises that displaced supracondylar fractures should receive early orthopaedic evaluation and, if indicated, timely surgical fixation [3]. Likewise, the AAOS clinical practice guidelines have generally recommended CRPP for all displaced Gartland type II and III fractures [4]. The emerging data suggest that within the framework of these guidelines, there may be a subset of ‘displaced’ type II fractures that can be managed successfully without surgery. However, before any official recommendations are changed, robust prospective evidence will be required to delineate and support the specific criteria that can identify these cases. In the meantime, clinicians should continue to use careful judgement on a case-by-case basis, incorporate the radiographic insights from recent studies, and ensure families are fully informed when considering a non-operative approach for these injuries.

## Conclusions

Non-operative management with closed reduction and casting appears to be a safe and effective option for a select subset of Gartland type II supracondylar fractures. This contrasts with currently available guidelines (BOAST and AAOS), which mostly favour operative management for type II fractures. However, the current evidence consists predominantly of small, retrospective studies that may be subject to selection bias. It is not yet robust enough to provide standardised criteria for determining which fractures can be safely managed non-operatively. Large, prospective, multicentre studies are required to validate radiographic thresholds, standardise subclassification and develop formal guidance on non-operative management, at which point alterations to existing guidelines could be considered.
